# Moderators of metabolic threshold adaptations: a systematic review and meta-analysis

**DOI:** 10.7717/peerj.21806

**Published:** 2026-10-22

**Authors:** Ratko Peric, Zoran Nikolovski, Dario Vrdoljak, Antonela Karmen Ivisic, Isaac A. Chavez-Guevara, Jara Vriese, Carlo Ferri Marini

**Affiliations:** 1 Department of Exercise Physiology, Orthopedic Clinic OrthoSport Banja Luka, Banja Luka, Bosnia and Herzegovina; 2 Faculty of Kinesiology, University of Split, Split, Croatia; 3 High Performance Sport Centre, Croatian Olympic Committee, Zagreb, Croatia; 4 Centre for Heart, Lung and Vascular Health, University of British Columbia, Okanagan Campus, Kelowna, Canada; 5 Faculty of Sports Ensenada, Universidad Autónoma de Baja California, Baja California, Mexico; 6 Department of Human Movement Sciences, University Medical Center Groningen, University of Groningen, Groningen, Netherlands

**Keywords:** Aerobic threshold, Anaerobic threshold, Training intensity, Dose–response relationship, Meta-analysis

## Abstract

Optimal training intensity relative to individual metabolic thresholds remains ambiguous despite its fundamental importance in endurance sports. The present pre-registered systematic review and meta-analysis (PROSPERO: CRD420251020702) evaluated the relative influence of potential moderators on adaptations in the aerobic (AeT) and anaerobic (AnT) thresholds. The primary outcome variable was the magnitude of change in physiological threshold values from pre- to post-intervention, quantified through power output (W), speed (km/h), or oxygen uptake metrics (VO_2max_). Three-level meta-analytic models with cluster-robust variance estimation quantified Log Response Ratio effect sizes across 13 studies (24 intervention groups, 338 participants). Among the substantive moderators examined (*i.e.*, training intensity, participant status, and intervention length), training intensity relative to the metabolic threshold showed the most consistent association with changes in threshold-related outcomes. For AeT (*k* = 9), a significant effect of training intensity was observed (*p* = 0.017), with supra-threshold training showing the largest improvement (+38.7%) compared with at-threshold (+14.7%) and sub-threshold (+18.4%) training. For AnT (*k* = 15), a significant categorical effect of training intensity was observed (*p* = 0.008), with *post-hoc* comparisons revealing greater adaptations for supra-threshold *versus* sub-threshold training (*p* = 0.010), alongside a graded descriptive pattern across categories (below: +2.6%, at: ∼+7%, above: +12.2%). However, substantial heterogeneity persisted (AeT: I^2^ ≈ 89%; AnT: I^2^ ≈ 53%), likely reflecting threshold identification bias (*e.g.*, lactate *vs.* gas analysis) and varying background training distributions. These findings identify training intensity as a primary moderator associated with threshold adaptations within the current literature, with supra-threshold metabolic range consistently outperforming at- or below-threshold prescriptions within the limited supra-threshold ranges represented in the included studies. Therefore, individualized, supra-threshold training with regular reassessment seems to be recommended to optimize individual training responses. However, substantial heterogeneity and methodological variability warrant caution, and further well-controlled studies are needed before definitive training recommendations can be made.

## Introduction

To meet the metabolic demands of physical activity, the human body relies on three integrated energy systems: oxidative, glycolytic, and phosphagen (Gastin, 2001). As exercise intensity increases, the relative contribution of these systems shifts, giving rise to distinct intensity domains commonly known as moderate, heavy, and severe (Skinner & McLellan, 1980; Poole & Jones, 2012). Two metabolic thresholds serve as the physiological delimiters between these intensity levels (Binder et al., 2008; Meyler et al., 2025). The first threshold, commonly termed the aerobic threshold (AeT), represents the upper boundary of predominantly oxidative metabolism and marks the transition from the moderate to the heavy intensity domain, where blood lactate demonstrates a first systematic rise above baseline values (Meyer et al., 2005). In contrast, the second threshold, termed anaerobic threshold (AnT), marks the transition from heavy to the severe intensity domain and represents the highest workload at which lactate production and clearance remain in equilibrium (Faude, Kindermann & Meyer, 2009; Brooks, 2018). Crucially, we recognise the wide range of terminology (Binder et al., 2008; Faude, Kindermann & Meyer, 2009) but for this synthesis, the first and second metabolic transitions are represented by the aggregate descriptors AeT and AnT, respectively.

Metabolic thresholds are increasingly conceptualized as dynamic transition regions rather than discrete, fixed intensities (Londeree, 1997), influenced by accumulating fatigue and metabolic stress (Pethick, Winter & Burnley, 2021; Burnley, 2022; Nuuttila et al., 2025). In this context, threshold-anchored intensity prescription represents a cornerstone of standardization (Stöggl & Sperlich, 2015), ensuring that the training stimulus is matched to an individual’s metabolic boundaries (Wolpern et al., 2015; Iannetta et al., 2020). Level 1a evidence demonstrates that threshold-based training produces superior improvements in aerobic capacity when compared with traditional, arbitrary intensity anchors (Meyler et al., 2025). AeT-anchored workloads enhance mitochondrial density, capillarization, and fat oxidation, whereas AnT-anchored intensities optimize lactate shuttling and buffering (Stöggl & Sperlich, 2015; Brooks, 2018). Governed by exercise intensity (MacInnis & Gibala, 2017), these adaptations arise from signaling pathways that upregulate mitochondrial biogenesis and lactate-shuttle function, concurrently raising thresholds and recalibrating the three energy systems (Holloszy & Coyle, 1984; Storoschuk et al., 2025). The resulting adaptations are strongly influenced by exercise intensity, highlighting the importance of accurately characterizing the intensity at which training is prescribed (MacInnis & Gibala, 2017). Training Intensity Distribution (TID) frameworks are therefore used to quantify this training ‘dose’ (Seiler & Tønnessen, 2009).

However, the conventional three-zone model exhibits limitations, as it aggregates metabolically heterogeneous workloads, from moderate to severe efforts, within broad categories (Seiler, 2010; Watts et al., 2023; Rivera-Köfler et al., 2025). Treating thresholds solely as fixed delimiters limits the ability to target the specific intensity ranges required for domain shifts (Watts et al., 2023; Meixner et al., 2025). Therefore, more granular multi-zone systems (*e.g.*, five-zone models) are used to resolve physiologically meaningful variation and identify intensity-specific dose–response relationships (Haugen et al., 2022; Watts et al., 2023; Sun et al., 2025).

Even within granular frameworks, prescriptions for threshold optimization remain disparate. For the AeT, some advocate for intensities below the anchor (*e.g.*, 90% AeT) to promote structural remodeling, maintaining minimal autonomic fatigue (Esteve-Lanao et al., 2007; Seiler & Tønnessen, 2009). Others support a dual-intensity approach (*e.g.*, 90%–110% AeT), addressing broader metabolic requirements (Stöggl & Sperlich, 2015). Regarding the AnT, some prioritize training precisely at the anchor (100%) to maximize lactate clearance (Manunzio et al., 2016), whereas others argue that only intensities in the severe domain (*e.g.*, ≥ 110%) provide sufficient stress for adaptation (Laursen et al., 2005; Casado et al., 2023). Ultimately, recommendations for optimal intensity remain vague due to persistent inconsistencies in the literature (Londeree, 1997; Faude, Kindermann & Meyer, 2009). Although prescribing training relative to individual thresholds may enhance performance (Faude, Kindermann & Meyer, 2009), chronic work at or near these zones raises concerns about long-term effectiveness and non-functional overreaching in elite athletes (Guellich & Seiler, 2010; Hofmann & Tschakert, 2017). Additionally, although empirically untested, this optimal intensity relative to the threshold may differ by fitness level, with less-trained individuals potentially benefiting from below-threshold stimuli and more highly trained athletes requiring greater supra-threshold exposure (Londeree, 1997; Seiler & Tønnessen, 2009). While anchoring intensity to thresholds is recognized as an effective method (Meyler et al., 2025; Sun et al., 2025), a one-size-fits-all approach may not account for the dynamic nature of these boundaries (Subbarayalu et al., 2024). Moreover, the influence of training and participant characteristics on threshold adaptation remains unclear (Seiler & Tønnessen, 2009; Casado et al., 2023).

Therefore, the purpose of this systematic review and meta-analysis is to evaluate how training and participant characteristics are associated with metabolic threshold adaptations, bridging the gap between physiological theories and coaching practice. Specifically, the present study aims to: (i) evaluate how moderators influence adaptations in the AeT and AnT; (ii) identify the primary moderator of threshold adaptation; and (iii) characterize the variability of threshold responses across individuals and training conditions.

## Methodology

### Search strategy

This study was prospectively reviewed and registered on the PROSPERO platform under registration number CRD420251020702. In accordance with the PRISMA guidelines (Moher et al., 2009), two investigators (RP & ZN) independently carried out literature search across PubMed & Google Scholar databases. To ensure the comprehensiveness of the search strategy, we also performed ‘backward snowballing,’ manually reviewing the reference lists of all included studies and previous systematic reviews to identify any papers not captured by the electronic search string. The search period spanned from the inception of each database until July 2025. Boolean logic keywords were systematically applied to combine English search terms across four domains: “Aerobic Threshold” OR “Anaerobic Threshold” OR “Lactate Threshold” OR “Ventilatory Threshold” OR “Maximal Lactate Steady State” OR “Gas Threshold” AND “Exercise” OR “Training” OR “Physical Activity” AND “Intensity” OR “Duration” OR “ Frequency” OR “Length” AND “Athletes” OR ”Amateur”. The search terms “Lactate Threshold” OR “Ventilatory Threshold” were utilized as a comprehensive descriptor to capture studies reporting on both the first and second threshold, which are frequently referred to in the literature under different names, respectively (Meyer et al., 2005). By restricting the search to English-language peer-reviewed literature, we aimed to minimize the risk of misinterpreting technical identification markers (*e.g.*, specific lactate mM fix points or gas exchange slopes) that could arise from translation errors, thereby preserving the internal validity of the meta-analytic models.

### Study inclusion and exclusion criteria

Eligibility criteria for inclusion were defined using the PICOS framework (Population, Intervention, Comparison, Outcome, Study design) (Huang, Lin & Demner-Fushman, 2006). Population: Healthy adults (both sexes), aged 18–60 years. Intervention: Studies where training intensity was normalized to individual metabolic thresholds. The specific method of threshold determination (*e.g.*, lactate (LT) or ventilatory (VT) analysis) was not restricted, provided the method was consistent within the study. Inclusion was not restricted to work-matched protocols; instead, session duration and frequency were extracted as independent moderators among the variables explored to evaluate the relative influence of total training volume *versus* intensity. Comparison: Pre- *versus* post-intervention threshold values. Outcomes: Quantifiable pre-to-post changes in threshold values. These metrics included external measures (*e.g.*, power output (W) or running speed (km/h)) or internal measures (*e.g.*, VO_2max_) recorded at a predefined physiological criterion such as a LT or VT. As such, extracted outcomes reflect changes in the workload or physiological response corresponding to the identified threshold, rather than direct measurement of the underlying metabolic transition point itself. Study Design: Randomised controlled trials (RCTs), non-randomized controlled trials (CTs), and single-group pre-post interventions. Case reports, conference abstracts, reviews, letters, opinion pieces, and non-English publications were excluded.

### Data extraction

Four independent reviewers (RP, DV, AI and ZN) conducted an initial screening of the identified literature by reviewing duplicates, language, title, and abstracts. After a first round of revision, included studies were assessed for their full-text eligibility. Thereafter, manual screening of the remaining studies and data extraction was carried out independently by two researchers (RP & JHV), who remained blinded to minimise bias. When data were unavailable in the original papers, authors were contacted. Pre- and post-intervention means and standard deviations (SD) were extracted into a pre-customised sheet, including: Study characteristics (authors, year), Participant demographics (sample size, sex, age, training status (untrained, amateur, elite)), Threshold identification method (LT) or (VT) and type (AeT and AnT), Intervention details (exercise modality (*e.g.*, cycling, running), training format (*e.g.*, continuous, interval), session duration (minutes), frequency (sessions/week), total length (weeks), relative intensity (below, at, above expressed as % of threshold (if the % of the threshold was not reported, it was calculated from the available data)), and intensity reporting metrics at baseline and post-intervention (*e.g.*, km/h, W, or relative (%) and absolute (L/min & ml/min/kg) VO_2max_). If studies reported multiple outcome metrics for the same threshold, all parameters were extracted. However, to avoid unit-of-analysis errors and adhere to the assumption of independent observations, a single primary outcome was selected for analysis based on a predefined hierarchical rule (Higgins et al., 2024), prioritizing the most direct and prescriptively actionable performance metric.

For this study, the terms ‘sub-threshold’ and ‘supra-threshold’ indicate exercise intensities positioned below (<100%) and above (>100%) the identified physiological anchor (AeT or AnT), respectively. The magnitude of deviation below or above 100% is not fixed a priori; rather, one of the aims of this review is to synthesize existing evidence to characterize the typical width and variability of the threshold zones across individuals and training conditions. By anchoring analyses to these intensity bands, we aim to capture subtle differences in adaptive stimulus and the dynamic boundaries of metabolic adaptation. Since all interventions targeted threshold-based training, intensity classification reflected only the relative position of the prescribed sessions with respect to the threshold (sub-, at-, or supra-), rather than a comparison across different intensity domains. The metrics used for statistical analysis were summarised in Table 1.

**Table 1:** Summary of all included studies with LnRR values back-transformed into percentage changes.

**Study**	**N**	**Sex**	**Age**	**Status**	**Modality**	**Format**	**Length (wks)**	**Frequency (wk)**	**Duration (min)**	**Threshold ID method**	**Threshold type**	**Category**	**Relative intensity (%)**	**Outcome unit**	**Pre mean**	**Pre SD**	**Post mean**	**Post SD**	**% Change**
Inglis et al. (2024)	14	M/F	25	Amateur	Cycling	Continuous	6	3	50	VT	AeT	BELOW	90	PO (W)	87	27	103	27	18.4
	14	M/F	27	Amateur	Cycling	Continuous	6	3	41	VT	AeT	ABOVE	110	PO (W)	93	22	119	27	28.0
	14	M/F	26	Amateur	Cycling	Continuous	6	3	30	VT	AnT	AT	100	PO (W)	136	35	156	32	14.7
	14	M/F	27	Amateur	Cycling	Interval	6	3	Varied	VT	AnT	ABOVE	120	PO (W)	123	45	152	48	23.6
	14	M/F	28	Amateur	Cycling	Interval	6	3	Varied	VT	AnT	ABOVE	150	PO (W)	137	36	153	39	11.7
Weltman et al. (1992)	9	F	31.3	Untrained	Running	Continuous	52	6	Varied	LT	AeT	AT	100	Speed (m/min)	112.2	8.3	138.9	20.3	23.8
	8	F	31.3	Untrained	Running	Mixed	52	6	Varied	LT	AeT	ABOVE	127	Speed (m/min)	121.2	24.7	170.0	13.1	40.3
Henritze et al. (1985)	12	F	21.8	Untrained	Cycling	Continuous	12	5	50	LT	AeT	AT	100	VO_2_ (mL kg^−1^ min^−1^)	18.3	3.5	21.6	6.2	18.0
	11	F	21.8	Untrained	Cycling	Continuous	12	5	35	LT	AeT	ABOVE	147	VO_2_ (mL kg^−1^ min^−1^)	16.5	5.7	24.4	6.6	47.9
He et al. (2008)	102	M	19	Untrained	Running	Continuous	18	3	Varied	VT	AeT	AT	100	VO_2_ (L min^−1^)	2.83	0.28	2.89	0.36	2.1
Enoksen, Shalfawi & Tønnessen (2011)	10	M	19.9	Amateur	Running	Continuous	10	6	Varied	LT	AnT	BELOW	84	Speed (km/h)	15.3	0.8	15.7	0.7	2.6
	9	M	19.9	Amateur	Running	Continuous	10	6	Varied	LT	AnT	AT	100	Speed (km/h)	14.6	1.0	15.2	0.8	4.1
Hebisz & Hebisz (2024)	12	F	17.9	Amateur	Cycling	Continuous	8	3.5	165	VT	AeT	AT	100	PO (W)	128.4	22.9	138.5	22.8	7.9
	12	F	17.9	Amateur	Cycling	Interval	8	3.5	Varied	VT	AnT	ABOVE	119	PO (W)	195.7	39.8	203.0	42.6	3.7
	12	F	17.9	Amateur	Cycling	Continuous	8	3.5	165	VT	AeT	AT	100	PO (W)	129.0	14.8	157.1	22.6	21.8
	12	F	17.9	Amateur	Cycling	Interval	8	3.5	Varied	VT	AnT	ABOVE	119	PO (W)	197.7	28.8	232.3	23.0	17.5
Czuba et al. (2018)	10	M	20.5	Amateur	Cycling	Continuous	4	3	65	LT	AnT	AT	100	PO (W)	292	21.4	306	16.4	4.8
Tota et al. (2019)	15	M	NR	Elite	Running	Mixed	14	8	Varied	VT	AnT	ABOVE	124	Speed (km/h)	11.0	0.6	12.0	0.4	9.1
Dufour et al. (2006)	9	M	30.3	Amateur	Running	Continuous	6	2	32	VT	AnT	AT	100	Speed (km/h)	17.2	0.4	17.8	0.4	3.5
Keith, Jacobs & McLellan (1992)	8	M	29.9	Amateur	Cycling	Continuous	8	3	30	LT	AnT	AT	100	%VO_2max_	70.5	8.2	79.8	5.9	13.2
	7	M	31.0	Amateur	Cycling	Interval	8	3	30	LT	AnT	AT	100	%VO_2max_	71.1	12.4	80.7	9.1	13.5
Tanaka et al. (1984)	21	M	18.5	Amateur	Running	Mixed	36	6	Varied	LT	AnT	AT	100	Speed (km/h)	14.7	1.3	15.0	1.4	2.0
Denis et al. (1982)	5	M	35	Amateur	Cycling	Continuous	40	3	60	Both	AnT	ABOVE	112	%VO_2max_	74	4	81	4	9.5
Hurley et al. (1984)	8	M	29	Untrained	Mixed	Mixed	12	6	Varied	LT	AnT	ABOVE	129	%VO_2max_	68	4	75	3	10.3

**Notes.**

M Males F Females wks Weeks wk Weekly LT Blood lactate VT Gas analysis AeT Aerobic threshold AnT Anaerobic threshold SD Standard deviation Mixed both continuous and interval training PO Power output VO_2_ Oxygen uptake VO_2max_ Maximal VO_2_

Mixed format intensity was categorized based on average intensity relative to the threshold. Interval training included both high intensity interval training (HIIT) and sprint interval training (SIT).

### Risk of bias assessment

Two reviewers (RP and JHV) independently assessed the risk of bias, with disagreements resolved by a third reviewer (CFM). The assessment tool was selected based on study design: For RCTs: The Cochrane Risk of Bias 2 (RoB 2) tool (Sterne et al., 2019) evaluated bias across five domains: randomisation process, deviations from intended interventions, missing outcome data, outcome measurement, and selective reporting. Overall judgments were classified as “Low,” “Moderate,” or “High” risk of bias. For Non-Randomised Studies: The ROBINS-I tool (Sterne et al., 2016) was used for prospective non-randomised, pre-post, and longitudinal intervention studies. It assessed bias across seven domains, including apart from abovementioned, confounding and selection of participants. Overall risk was categorised as “Low,” “Moderate,” “Serious,” and “Critical.” For this review, “Serious” and “Critical” risk levels were grouped as “High” risk to provide a standardized quality classification across assessment tools, as both categories signify findings with a high risk of bias that limits evidence certainty (Sterne et al., 2016).

### Statistical analysis

All statistical analyses were performed in R (version 4.3.2; R Core Team, 2023) using the dplyr package (Wickham et al., 2023), metafor package (Viechtbauer, 2010), and the robumeta package (Fisher & Tipton, 2015). A two-tailed *p*-value of less than or equal to 0.05 was considered statistically significant for all inferential tests. The primary outcome variable was the change in metabolic threshold values from pre- to post-intervention. To address the known limitations of raw percent change (Nakagawa & Cuthill, 2007), effect sizes (ESs) for each intervention group were calculated as the Log Response Ratio (LnRR), following Hedges, Gurevitch & Curtis (1999). The sampling variance for each LnRR was calculated using pre- and post-intervention standard deviations (SD), sample sizes, and an assumed pre-post correlation of *r* = 0.7 (Lajeunessei, 2011). This enabled formal inverse-variance weighted meta-analyses, in which each intervention group contributes proportionally to its precision (Borenstein et al., 2009).

All analyses were stratified by threshold type, AeT and AnT (Meyer et al., 2005). To account for studies contributing multiple intervention groups, a three-level meta-analytic model was used, partitioning variance into sampling (Level 1), within-study (Level 2), and between-study (Level 3) levels (Assink & Wibbelink, 2016). The magnitude of heterogeneity was quantified using the Q-statistic and the I^2^ index (Higgins et al., 2024). In meta-regression models, total heterogeneity was partitioned into the variance explained by moderators (QM) and the residual unexplained heterogeneity (QE), the latter testing whether significant between-study variability remained after accounting for moderators (Higgins et al., 2024). To illustrate the practical implications of the observed heterogeneity, 95% prediction intervals (PI) were also calculated for the overall pooled effects (Borenstein et al., 2009). Additionally, cluster-robust variance estimation (RVE) with small-sample corrections was applied (Hedges, Tipton & Johnson, 2010).

The analytical process involved three distinct stages. First, to inform variable selection, a comprehensive descriptive synthesis was conducted on all potential moderators which were subdivided into three groups: Intervention-level moderators: training intensity relative to threshold (sub-, at or supra-), training format (continuous *vs.* interval), intervention frequency (sessions per week), session duration (minutes per session), intervention length (weeks (wks)), exercise modality (running, cycling, mixed); Participant-level moderators: participant training status (untrained, amateur, elite), participant sex (male, female); and Methodological-level moderators: method of threshold determination (LT *vs.* VT), and outcome units (power, speed, and VO_2max_). In the context of this meta-regression, ‘training intensity’ is investigated as the primary intervention-level moderator. While intensity served as the independent variable in the included primary studies, in the meta-analytic framework it is treated as a moderator to evaluate its influence on the variance observed in effect sizes across the 24 intervention groups. Other variables (*e.g.*, duration, frequency) are treated as secondary moderators to evaluate their potential to alter the strength of the intensity-adaptation relationship. To prevent unit-of-analysis errors, an a priori hierarchy of outcome units was established based on performance prescriptive relevance rather than physiological primacy (power > speed > VO_2max_), ensuring that only the single highest-ranked outcome was extracted from any study group reporting multiple measures (Higgins et al., 2024). Power output was prioritized as the most direct measure of external work, particularly in cycling-based studies, which comprised the majority of our sample, whereas speed was prioritized over VO_2__max_ because it represents an actionable external performance metric (Impellizzeri, Marcora & Coutts, 2019). In contrast, although VO_2__max_ is an important physiological indicator, it is less commonly used as the primary metric for field-based intensity prescription because it represents an internal physiological response and is susceptible to daily biological variation (Meyler, Bottoms & Muniz-Pumares, 2021). This exploratory step aimed to identify the most prominent physiologically relevant moderators for formal meta-regression by assessing their signal strength, calculated as the difference between the highest and lowest Mean % Change within each moderator (max–min). Moderators with larger differences were prioritised, providing preliminary evidence of potential influence on heterogeneity (Higgins et al., 2024). This stage was an exploratory descriptive prioritization procedure, not statistical evidence that a moderator was important. Participant training status was accepted as defined by the studies whereas amateurs included both active and well-trained athletes. Based on signal strength and established theoretical relevance, three substantive moderators (training intensity, participant status, and intervention length) were ranked for formal modelling to reduce the risk of overfitting. The analytical approach for training intensity was adapted to the data structure of each subgroup: it was treated as a categorical variable (below, at, above) for both the AeT and AnT subgroups to compare group-level effects. For the AnT subgroup, the ordered nature of these categories additionally allowed for a descriptive assessment of the adaptive gradient across the sampled intensity levels. For the formal analysis, the participant’s training status was retained despite its relatively small effect, due to its established theoretical relevance: baseline fitness influences the magnitude of training-induced threshold shifts (Jones & Carter, 2000). Including training status allowed potential confounding from prior training background to be examined, even with limited variability in the sample.

Second, a primary moderator analysis was performed by fitting separate, single-predictor meta-regression models for each of the three substantive moderators using cluster-RVE. This unified approach was used to test for significant moderation *via* omnibus test of moderators (QM, *p* < .05) while handling both categorical and continuous data (Higgins et al., 2024). For categorical moderators, the model functioned as a formal subgroup analysis, comparing effects across distinct levels. For continuous moderators, it tested for a significant linear trend.

Third, a methodological bias assessment was conducted. For this, a series of ANCOVA-style meta-regression models were fitted to assess the influence of two key methodological variables (threshold determination method and outcome unit). This was achieved by adding each variable as a predictor to a model that already statistically controlled for the primary substantive moderators (Higgins et al., 2024). Given the limited sample size, particularly for the AeT subgroup (*k* = 9), these analysis of covariance (ANCOVA) models were interpreted as hypothesis-generating rather than confirmatory.

Model validity was examined through comprehensive *post-hoc* diagnostics, including evaluation of residuals for linearity, homoscedasticity, independence (Durbin & Watson, 1951), and normality (Shapiro & Wilk, 1965). Influential observations were identified using Cook’s distance (Cook, 1977). These comprehensive diagnostic procedures were applied to all meta-regression models in this study to ensure robust and interpretable results.

To account for dependent ESs within studies, we employed a cluster-robust bootstrap approach (2,000 iterations) that resampled entire studies with replacement, thereby preserving within-study dependencies (Efron & Tibshirani, 1994). Random-effects meta-regression was applied to each bootstrap sample to provide valid inference, particularly useful for avoiding computational instability inherent in resampling three-level models (Hedges, Tipton & Johnson, 2010; Tipton, Pustejovsky & Ahmadi, 2015). This sensitivity analysis focused specifically on intervention length as a continuous moderator, given its consistent reporting across studies, theoretical importance for understanding training time-course effects, and to maintain adequate statistical power given our sample size constraints. All primary inferential statistics, including regression coefficients and their 95% CI, were derived from the main multilevel models using cluster RVE with small-sample corrections, explicitly accounting for within-study dependencies, as they fully convey the precision and statistical significance of the estimates without needing to report standard errors (Higgins et al., 2024).

Sensitivity analyses included leave-one-study-out (LOSO) testing to ensure that no single study disproportionately influenced the conclusions (Higgins et al., 2024). Publication bias was assessed *via* visual inspection of funnel plots and formally tested using Egger’s regression test (Egger et al., 1997).

Because of the limited sample sizes, the precision of estimated effects was evaluated primarily through inspection of CI width, consistent with recommendations for small-sample meta-analytic inference (Tipton, Pustejovsky & Ahmadi, 2015). Weighted regression estimates were reported alongside unweighted descriptive summaries for context, providing both precision-weighted and raw group-level perspectives (Borenstein et al., 2009).

**Figure 1:**
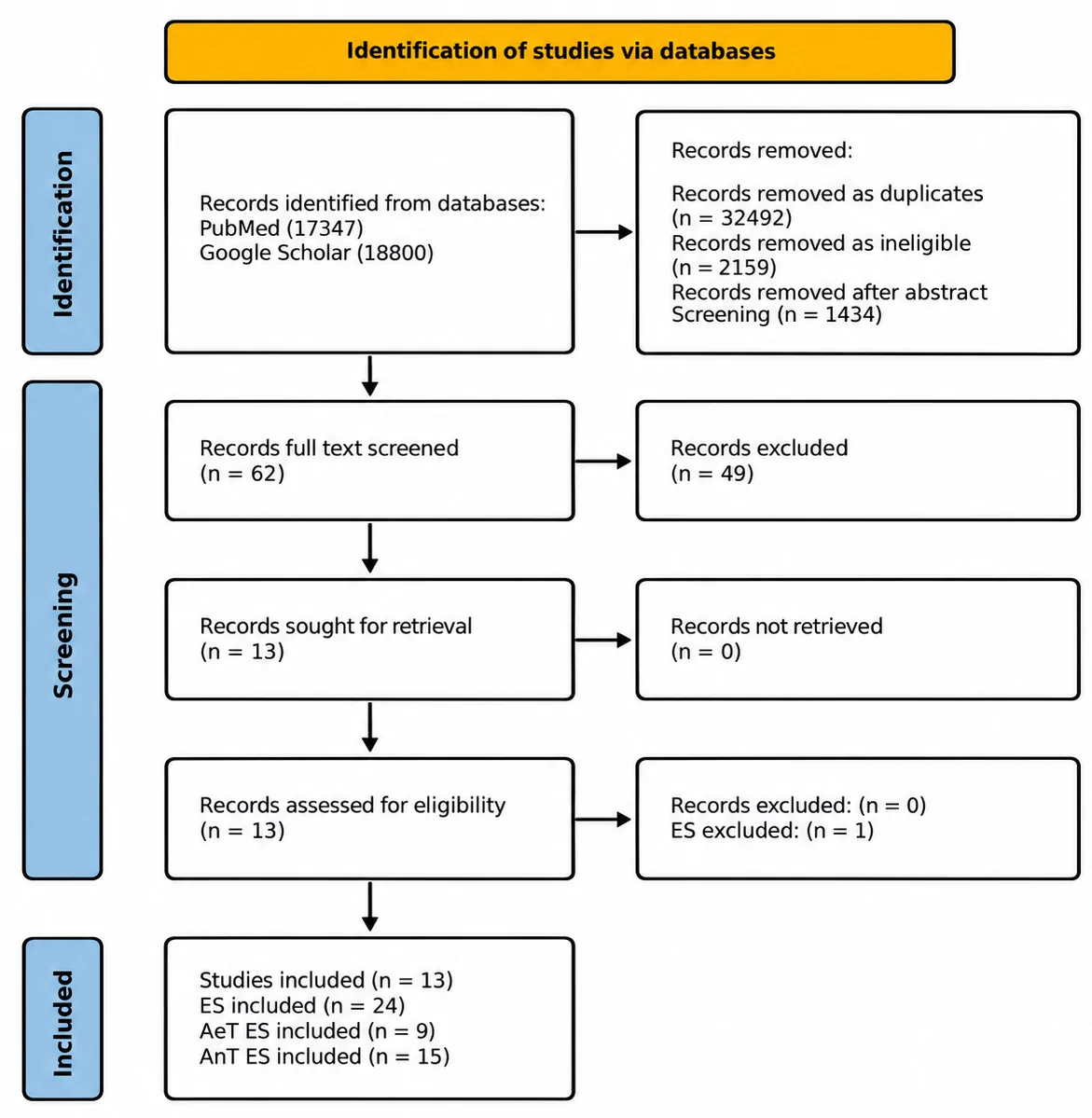
PRISMA flow diagram.

## Results

### Study selection and risk of bias

The systematic search retrieved 36,147 records from PubMed and Google Scholar. After eligibility analysis and duplicates removal, 1,496 titles and abstracts were screened. Of these, 62 full-text articles were cleared for eligibility with forty-nine studies further excluded for failing to meet inclusion criteria. This resulted in 13 studies included in the final analysis, as illustrated in the PRISMA flow diagram (Fig. 1). These 13 studies resulted in 25 unique intervention groups (k). However, one group was excluded from further analysis due to the study conditions being hypoxic (Czuba et al., 2018), eventually leaving 24 groups to be included in our study. A major methodological constraint shared by almost all of the 13 included studies was the absence of blinded outcome assessment. The methodological quality of the included studies varied, with acceptable risk of bias results (Supplemental Information File 1).

### Descriptive characteristics

The analysis included 338 participants (239 (71%) males, 99 (29%) females). The weighted mean age of participants was 25.8 ± 5.6 years. The included groups represented a wide spectrum of fitness levels: six groups (25.0%) were classified as untrained, seventeen (70.8%) as amateur and only one (4.2%) as elite. Groups were determined as reported by the authors. Regarding the primary outcome, 15 of the intervention groups (62.5%) assessed the AnT, while nine groups (37.5%) assessed the AeT. Threshold determination methods included LT (11 groups), VT (12 groups), and one study reported both. Outcome units were power (10 groups; 41.7%), speed (seven groups; 29.2%) and VO_2max_ (seven groups; 29.1%). Cycling was the predominant exercise modality (15 groups; 62.5%), followed by running (eight groups; 33.3%) and mixed-modality training (one group; 4.2%). Intervention length varied substantially, where short-term interventions (4–8 wks) included 13 groups (54.2%), medium-term interventions (10–18 wks) encompassed seven groups (29.2%) and long-term interventions (36–52 wks) incorporating four groups (16.7%). Training frequency varied from low (2–3/week; 11 groups (45.8%)) to moderate (3.5–5/week; six groups (25.0%)) and high (6–8/week; seven groups (29.2%)). Half of the studies implemented different training formats, including continuous (*k* = 4), interval (*k* = 4), and mixed-format (*k* = 4) groups. The remaining 12 (50%) groups used continuous format. Regarding the control of training load, 10 out of the 24 intervention groups (41.7%) used work-matched protocols to isolate the effect of intensity, while 14 groups (58.3%) followed protocols where both intensity and volume varied. A detailed description of the included studies with all LnRR values back-transformed into percentage changes is available in Table 1.

### Primary moderator analysis

Based on a systematic exploration (see Supplemental Information File 1), three substantive moderators (training intensity, participant status and intervention length) demonstrated the strongest and most consistent physiological signals across both subgroups, whereas others were not prioritised to prevent overfitting (Table 2).

**Table 2:** Unweighted descriptive statistics (percentage changes) and signal strength of primary moderators.

**Subgroup**	**Moderator**	**Level/intensity**	**k**	**Mean % change**	**Signal % strength**
AeT	Training intensity	Above	3	38.7	24.01
		At	5	14.7	
		Below	1	18.4	
	Participant status	Untrained	5	26.4	7.40
		Amateur	4	19.0	
	Intervention length	Long (≥ 36 wks)	2	32.1	30.85
		Medium (10–18 wks)	4	19.6	
		Short (4–8 wks)	3	23.7	
AnT	Training intensity	Above	7	12.2	9.60
		At	7	8	
		Below	1	2.6	
	Participant status	Untrained	1	10.3	1.20
		Amateur	13	9.8	
		Elite	1	9.1	
	Intervention length	Long (≥ 36 wks)	2	5.8	11.38
		Medium (10–18 wks)	3	9.9	
		Short (4–8 wks)	10	10.8	

**Notes.**

k number of groups wks weeks AeT aerobic threshold AnT anaerobic threshold Signal% strength difference between the highest and lowest mean % change within each moderator (max–min)

The overall pooled analysis for the AeT subgroup (*k* = 9) revealed a large and statistically significant training effect. The weighted mean improvement was +21.0% with 95% CI [11.8%–31.0%] (*p* < 0.001). The analysis suggested the presence of substantial heterogeneity between studies (*Q* = 39.17, *p* < 0.001; I^2^ ≈ 89%), indicating that true differences between interventions accounted for nearly 90% of the observed variance. This was further highlighted by the wide 95% PI, which ranged from −1.8% to +49.2%, suggesting that while the average effect is strongly positive, the outcome for a single future study could even be negative. To investigate the sources of this heterogeneity, a series of single-predictor meta-regression models using cluster-RVE were conducted. Training intensity was a significant moderator (QM (*df* = 2) = 8.18, *p* = 0.017), with results indicating that training at the AeT produced a significantly smaller effect than training above it (*p* = 0.046). However, a significant amount of residual heterogeneity remained even after accounting for intensity (QE = 17.58, *p* = 0.007), indicating that other factors also contribute to the results. In contrast to the descriptive exploration which suggested larger gains for untrained participants, the formal subgroup analysis for participant status did not find a statistically significant difference between untrained and amateur athletes (QM (*df* = 1) = 0.06, *p* = 0.802), and a large amount of heterogeneity remained unexplained by this moderator (QE = 39.14, *p* < 0.001). Likewise, the intervention length was also not a significant moderator (QM (*df* = 1) = 0.70, *p* = 0.404) and observed amount of heterogeneity remained unexplained by this moderator (QE = 32.98, *p* < 0.001).

For the AnT subgroup (*k* = 15), the overall pooled analysis also demonstrated a statistically significant, albeit smaller, training effect. The weighted mean improvement was +7.7% with 95% CI [4.9%–10.7%] (*p* < 0.001). Significant moderate heterogeneity was present in this subgroup (*Q* = 40.83, *p* = 0.002; I^2^ ≈ 53%). The 95% PI ranged from −0.3% to +16.4%, suggesting that while the average effect is positive, the effect in a future study could range from zero to substantive. The subsequent RVE meta-regression analysis revealed that a significant portion of the variance was explained by training intensity (QM (*df* = 2) = 9.59, *p* = 0.008). The model showed that training below the AnT resulted in significantly smaller adaptations than training above the AnT (*p* = 0.010), while the difference between training at and above the AnT showed only a non-significant trend (*p* = 0.054). After accounting for intensity, the amount of residual heterogeneity was no longer statistically significant (QE = 20.49, *p* = 0.058). While descriptive trends suggested larger improvements in untrained individuals for AnT, the formal subgroup analysis for participant status found no statistically significant difference between untrained, amateur, and elite groups (QM (*df* = 2) = 0.31, *p* = 0.858), with a large and significant amount of heterogeneity remaining unexplained by this moderator (QE = 35.39, *p* = 0.004). Similarly, the formal analysis of intervention length showed no statistically significant relationship with training outcomes (QM (*df* = 1) = 0.31, *p* = 0.579), with substantial residual heterogeneity present (QE = 40.82, *p* = 0.001). Even though the descriptive analysis identified participant status and intervention length as potential moderators based on mean differences, the formal inferential testing found no statistically significant moderation effects for either variable in either threshold domain, leaving only training intensity as the most dominant signal within the available data. Entire study data including master data table and raw results provided by R are available at https://doi.org/10.5281/zenodo.17523709.

### Assessment of methodological bias

For the AeT subgroup (*k* = 9), the ANCOVA model testing the threshold identification method (*i.e.,* VT or LT) as a potential bias source revealed significant overall moderator effects (QM = 22.73, *p* < 0.001) with no residual heterogeneity (QE = 4.13, *p* = 0.25). The threshold identification method demonstrated significant bias, with studies using VT reporting lower ESs compared to the LT method (β = −0.18, 95% CI [−0.33 to −0.03], *p* = 0.018). The model also suggested the importance of training intensity, as training at the threshold resulted in significantly smaller gains than training above it (β = −0.13, *p* = 0.008). The other controlled covariates, participant training status (*p* = 0.086) and intervention length (*p* = 0.88), were not statistically significant predictors of the outcome in this model. The ANCOVA model testing for potential bias source from outcome unit on the AeT (QM = 13.57, *p* = 0.035; QE = 3.75, *p* = 0.15) indicated a significant overall effect, with no significant residual heterogeneity remaining. The specific outcome units (VO_2max_: β = 0.33, 95% CI [−0.40 to 1.06], *p* = 0.37; speed: β = −0.75, 95% CI [−4.99 to 3.48], *p* = 0.73) did not show statistically significant independent effects compared to power. The training intensity category showed a significant effect, with supra-threshold training showing a higher effect (β = −0.13, 95% CI [−0.23 to −0.03], *p* = 0.008). Participant training status (β = −0.12, 95% CI [−0.25 to 0.02], *p* = 0.086), and intervention length had no effect (β = 0.003, 95% CI [−0.003 to 0.004], *p* = 0.88).

In contrast, the AnT subgroup (*k* = 15) ANCOVA model did not reveal any significant moderator effects (QM = 2.51, *p* = 0.87) between threshold identification methods, with residual heterogeneity remaining modest (QE = 20.16, *p* = 0.010). Specifically, the threshold identification method was not a source of bias, with neither LT (β = −0.06, 95% CI [−0.22 to 0.09], *p* = 0.40) nor VT (β = −0.04, 95% CI [−0.22 to 0.13], *p* = 0.62) showing a significant effect on the reported outcomes. None of the controlled covariates, relative training intensity (β = 0.0007, 95% CI [−0.001 to 0.003], *p* = 0.51), participant status (elite: β = −0.005, 95% CI [−0.12 to 0.11], *p* = 0.94; untrained: β = 0.02, 95% CI [−0.11 to 0.15], *p* = 0.76), or intervention length (β = −0.001, 95% CI [−0.005 to 0.003], *p* = 0.48), were significant predictors in this analysis. The ANCOVA model testing outcome unit bias in the AnT subgroup revealed significant overall moderator effects (QM = 17.02, *p* = 0.009) with non-significant residual heterogeneity (QE = 12.67, *p* = 0.12). Studies that used speed to measure performance reported significantly smaller ESs compared to those using VO_2max_ (β = −0.07, 95% CI [−0.13 to −0.01], *p* = 0.014). In contrast, using power as an outcome unit was not associated with a significant bias (power: β = −0.03, 95% CI [−0.10 to 0.04], *p* = 0.40). None of the controlled covariates, relative training intensity (β = 0.001, 95% CI [−0.001 to 0.003], *p* = 0.24), participant status (elite: β = 0.02, 95% CI [−0.05 to 0.10], *p* = 0.54; untrained: β = −0.04, 95% CI [−0.14 to 0.05], *p* = 0.38), or intervention length (β = −0.001, 95% CI [−0.003 to 0.001], *p* = 0.37), were significant predictors in this model. Overall, training intensity emerged as the most consistent moderator of training-induced changes in threshold-related performance outcomes, while participant training status, intervention length, threshold identification method, and outcome units had minimal impact.

### Model validation and sensitivity analysis

Model diagnostics (residual normality *via* Shapiro–Wilk, influential data points *via* Cook’s distance) and sensitivity analyses (LOSO and bootstrap) supported robust model estimates (Supplemental Information File 1). Nevertheless, publication bias was indicated for the AnT subgroup based on visual inspection of the funnel plot and Egger’s regression test (Supplemental Information File 1), suggesting that ESs may be overestimated. To assess the impact of our assumed pre-post correlation coefficient (*r* = 0.7), a sensitivity analysis was performed by varying the coefficient between 0.5 and 0.9. These alternative assumptions resulted in no meaningful changes to the pooled effect size estimates or the significance of the moderators, confirming that the findings are robust to the choice of correlation coefficient.

## Discussion

This systematic review and meta-analysis synthesised current evidence on exercise intensity matched to individual metabolic thresholds. Our results indicate that training intensity relative to the specific threshold is the most consistent moderator of threshold adaptation, whereas other moderators had made minimal to no contribution. Our findings reinforce the conceptualisation of metabolic thresholds as dynamic, individualised ranges rather than fixed points, a notion increasingly supported by evidence (Londeree, 1997; Binder et al., 2008; Nuuttila et al., 2025).

### Interpretation of key findings and moderator selection

The most consistent finding from our analysis was the association between training intensity relative to the metabolic threshold and greater improvements in threshold-related outcomes. The available evidence suggests that training above the identified anchors may produce greater threshold-related adaptations than training at or below them, aligning with the foundational intensity threshold concept for adaptation (Sady et al., 1980). This association was observed across both AeT and AnT analyses, although the available evidence does not establish an independent causal effect of intensity or identify an optimal supra-threshold intensity. Additionally, our analysis revealed a critical nuance for AnT. Specifically, the graded pattern of improvements across the below, at, and above categories—with supra-threshold training yielding approximately 4.7 times greater gains than sub-threshold work—suggests a continued dose–response relationship across the sampled intensities, although the formal inferential model was based on categorical comparisons (*df* = 2). This advises that adaptation magnitude is not a binary function of being above an anchor, but a graded response. Such finding refines traditional beliefs (Sady et al., 1980) and highlights the need to better define the intensity at which further increases cease to provide additional benefit.

#### AeT adaptations

For the AeT, meta-regression identified training intensity above the threshold as having the strongest association with adaptation (*p* = 0.017), producing a mean improvement of +38.7% compared to +18.4% for sub-threshold work. Weighted subgroup analysis suggested exercise intensity as the only significant moderator (*p* = 0.017). A plausible biological rationale for this superior adaptation (twofold) may involve pronounced disturbances in intramuscular homeostasis, including significant phosphocreatine depletion and elevated AMP/ATP ratios, which are minimal during moderate-intensity exercise (below AeT) (Storoschuk et al., 2025). Such metabolic stress robustly activates the PGC-1*α*/PPAR*δ* signalling axis, the master regulator of mitochondrial biogenesis, while upregulating fatty acid transport and oxidation enzymes (Holloszy & Coyle, 1984; Holloway et al., 2008; Egan & Zierath, 2013). This molecular response is specifically sensitive to exercise intensity, providing a biological rationale for the superior adaptations observed when training exceeds the metabolic threshold (MacInnis & Gibala, 2017). Concurrently, sustained VEGF signalling promotes capillary growth to enhance oxygen delivery (Brooks, 2018). While these structural adaptations are essential for maximizing cardiorespiratory fitness, they represent a high-cost stimulus; its implementation is constrained by rapid fatigue accumulation and the risk of non-functional overreaching (Guellich & Seiler, 2010). To maintain adherence and prevent autonomic burnout or overreaching, supra-threshold training should be viewed as a surgical tool, used strategically within a foundation of moderate-intensity volume (Guellich & Seiler, 2010).

It is important to emphasize that while supra-threshold training appears more effective for specifically shifting metabolic anchors, this does not imply that sub-threshold training is physiologically inferior in a global sense. Even though our findings challenge the conventional emphasis on exclusively high-volume, moderate-intensity sub-threshold exercise for improving the AeT (Seiler, 2010), sub-threshold training could elicit comparable shifts if sufficient volume is accumulated, a factor that was not directly quantified in this meta-analysis. However, such adaptations appear twofold less effective compared to supra-threshold training, which more strongly stimulates mitochondrial volume density, respiratory capacity, and lactate clearance; mechanisms related to a more potent threshold upward shift (Mølmen, Almquist & Skattebo, 2025). Furthermore, a sub-threshold AeT exercise may overlap with the intensity matching maximal fat oxidation (FAT_max_), which typically occurs below the AeT (Peric et al., 2022; Ferri Marini et al., 2023), highlighting its role in optimizing fat metabolism. In this way, sub-threshold training provides a metabolic and aerobic foundation that prepares the athlete to respond more effectively to supra-threshold training aimed at shifting AeT and improving threshold-related performance metrics (Watts et al., 2023). Therefore, high volumes of sub-threshold work remain essential for building an oxidative foundation without the excessive autonomic fatigue and prolonged recovery times associated with chronic supra-threshold stress (Seiler, 2010; Seiler & Tønnessen, 2009).

#### AnT adaptations

For the AnT, meta-regression supported a significant, positive graded dose–response relationship (*p* = 0.008) between prescribed relative intensity and AnT adaptation across the intensity range represented in the included studies. The descriptive data indicate a notable difference in adaptation magnitude: supra-threshold training elicited a +12.2% improvement, approximately 4.7 times more effective than sub-threshold work (+2.6%). These findings suggest that, within the supra-threshold intensity range examined, higher relative intensities were associated with greater improvements in AnT-related outcomes. This trend aligns with the hypothesized physiological requirement for repeated metabolic instability; exposing the muscle to intensities where lactate production transiently exceeds clearance triggers the recruitment of high-threshold type II motor units and the non-linear accumulation of metabolites (Bailey et al., 2009; MacInnis & Gibala, 2017). Such signals are the primary observed predictors for the upregulation of monocarboxylate transporters (MCT1 and MCT4) and the enhancement of intracellular buffering capacity (Meyer et al., 2005; Stöggl & Sperlich, 2015). Consequently, AnT optimization appears to follow a “higher is better” principle within the observed range of intensities, constrained primarily by fatigue management. Subtle supra-threshold stimuli allow time-in-zone near the metabolic boundary, whereas maximal stimuli provide concentrated metabolic flux required for rapid molecular remodelling and improved lactate shuttling (Stöggl & Sperlich, 2015; Muscella et al., 2020). This supports a graded adaptive response rather than a simple above–below threshold effect. Noteworthy, our findings challenge the notion that training precisely at the AnT is optimal (Manunzio et al., 2016); although the difference between training ‘at’ *versus* ‘above’ this anchor was marginally non-significant (*p* = 0.054), the point estimates and the overall dose–response relationship (*p* = 0.008) suggest that supra-threshold intensities provide a stronger stimulus. However, at-threshold training still outperformed sub-threshold prescriptions, indicating it retains adaptive value, although smaller than that observed with supra-threshold training. Importantly, because funnel-plot asymmetry and Egger’s test indicated potential small-study/publication bias for AnT, the magnitude of the pooled AnT effect, and potentially the intensity gradient, should be interpreted cautiously.

#### Comparative physiology of AeT and AnT

The contrasting supra-threshold adaptation patterns of the AeT and AnT reflect their distinct physiological roles. While AeT improvements yield a larger adaptive window and higher relative gains, AnT improvements produced smaller but more focused improvements. Although direct comparison of the relative magnitude of AeT and AnT adaptations should be interpreted cautiously, as percentage changes are influenced by the baseline magnitude of each threshold, the distinct adaptation patterns observed for AeT and AnT suggest that optimal intensity and modality are threshold-specific. Consequently, one-size-fits-all threshold models lack the necessary resolution for high-performance programming. To maximize adaptive efficiency, the AeT and AnT may need to be treated as distinct metabolic entities, each requiring independent and individualized training prescriptions tailored to their unique metabolic functions. The observed 2.7:1 ratio of AeT to AnT improvement magnitude reflects fundamental differences in the physiological scope of their respective adaptations (Bailey et al., 2009; Muscella et al., 2020; Mølmen, Almquist & Skattebo, 2025). A primary correlate of this disparity is the inherent ceiling effect imposed by maximal physiological capacities (VO_2max_ and HR_max_), with AnT upward shift confined to a narrower domain (Bailey et al., 2009; Brooks, 2018). Consequently, while AeT-anchored training may yield larger relative endurance gains due to this broader adaptive window, targeted AnT sessions remain essential for fatigue resistance and anaerobic capacity, despite their lower potential for large-magnitude percentage improvements.

### Moderator analysis and heterogeneity

Our evaluation prioritized three substantive moderators, training intensity, participant status, and intervention length, based on Stage 1 exploratory analysis. Formal meta-regression revealed exercise intensity as the only significant adaptive predictor for both thresholds, while participant status and intervention length showed descriptive trends but did not reach statistical significance. For participant status, the larger raw improvements in untrained subjects likely reflect their higher physiological ceiling for adaptation (Holloszy & Coyle, 1984). In contrast, for amateur and elite athletes, the same relative intensity represents a significantly higher absolute stress, elevating the risk of non-functional overreaching (Guellich & Seiler, 2010). Despite training intensity emerging as a significant moderator, substantial residual heterogeneity remained, particularly for AeT outcomes. This suggests that other unaccounted variables, such as individual training history, genetic predisposition, and nutritional status (*e.g.*, muscle glycogen availability), likely influence the magnitude of adaptation (Mann, Lamberts & Lambert, 2014). Furthermore, as training intensity is inherently intertwined with total volume and interval structure, our findings should be viewed as identifying a key association rather than establishing a sole causal mechanism (MacInnis & Gibala, 2017). It is possible that the observed superiority of supra-threshold training reflects a higher total metabolic stress rather than intensity alone, as several studies in our sample did not strictly work-match the sub- and supra-threshold protocols. Additionally, the ‘testing effect’, where participants improve their threshold scores simply by becoming more familiar with the physiological strain of the testing protocol (Hopkins, 2000), cannot be entirely ruled out in single-group designs. Future research using work-matched, randomized designs is necessary to isolate the independent effect of intensity from total energy expenditure.

Despite these differences in adaptive capacity, the dominance of the intensity stimulus suggests that the principle of supra-threshold adaptation appears broadly consistent across the populations examined (Londeree, 1997). Furthermore, intervention length (4–52 weeks) was not a significant predictor of adaptation magnitude, implying that the quality and potency of the stimulus (*i.e.,* intensity) may be more closely associated with threshold adaptations than the cumulative program duration. This aligns with evidence of rapid molecular remodelling following high-intensity stimuli (MacInnis & Gibala, 2017), though it does not negate the importance of long-term periodization for peak performance (Seiler & Tønnessen, 2009). Ultimately, the disconnect between the descriptive patterns and formal models for status and length likely stems from the confounding influence of limited sample sizes rather than a lack of physiological relevance.

#### Sources and implications of heterogeneity

Formal subgroup and meta-regression analyses successfully identified training intensity as a significant source of heterogeneity for both thresholds. In the context of exercise physiology, the high between-intervention heterogeneity observed in the AeT subgroup indicates substantial variability in the magnitude of effects across the included interventions. This heterogeneity may reflect a combination of differences in participant characteristics, training prescription, background training, threshold determination, outcome measurement, and genuine inter-individual differences in training responsiveness. Although responder/non-responder phenomena are well recognized in exercise training, the present meta-analysis cannot determine the extent to which the observed between-intervention heterogeneity reflects true individual differences in responsiveness (Egan & Zierath, 2013). Nevertheless, the presence of substantial heterogeneity supports the value of individualized training prescription rather than assuming that a single threshold-based stimulus will produce a uniform response across athletes. Furthermore, the lack of standardized reporting for background training volume (non-experimental sessions) likely contributed to the observed heterogeneity. If studies differed in the amount of moderate-intensity work performed alongside the prescribed threshold sessions, the total cumulative stress would vary, potentially confounding the magnitude of the threshold shift.

Methodological differences in threshold identification appear to be a secondary source of heterogeneity. While we utilized these markers as aggregate descriptors of metabolic transitions, we acknowledge that LT and VT do not identify identical physiological points. LT reflects the systemic net balance between lactate production and clearance, whereas VT represents a downstream, integrated pulmonary response to systemic CO_2_ buffering (Brooks, 2018; Binder et al., 2008). Our ANCOVA model confirmed this discrepancy in the AeT subgroup, where VT-based identification yielded significantly lower ESs compared to LT-based methods (*p* = 0.018). This lack of physiological ‘synchronicity’ likely accounts for a substantial portion of the residual heterogeneity (I^2^≈89%) observed, suggesting that the choice of determination method is not merely a technicality but a source of meaningful variance in recorded adaptations. Moreover, the lack of standardisation in LT determination (with more than 25 existing methods) likely amplifies these differences, potentially inflating ESs variability compared to the more standardised VT determination methods (Faude, Kindermann & Meyer, 2009; Chavez-Guevara et al., 2025).

For the AnT subgroup, the outcome unit was a significant moderator, with performance-based outcomes associated with lower effects (*p* = 0.014). Power and speed are external output measures that may be biased compared to internal output measures (*e.g.*, HR or VO_2max_), which directly quantify the physiological cost and are more suitable for evaluating adaptability (Boulay et al., 1997). Therefore, it would seem that the choice of outcome unit might affect the apparent amount of training benefits; hence, performance-based approaches might not adequately account for the underlying physiological changes compared to physiology–based methods (Impellizzeri, Marcora & Coutts, 2019).

Beyond measured factors, variables such as genetic predisposition or daily nutritional status (*e.g.*, muscle glycogen) are known to influence adaptation (Egan & Zierath, 2013; Chávez-Guevara et al., 2024) but were not considered in our study.

### Practical implications

Our findings suggest that, when the goal is to shift metabolic thresholds, supra-threshold work may play a more important role than traditionally emphasized, particularly when embedded within a broader intensity framework. Our findings advocate for a shift from targeting fixed threshold points to leveraging dynamic supra-threshold zones, recognizing inter-individual variability and fatigue-induced shifts (Binder et al., 2008; Nuuttila et al., 2025). For the AeT, this zone provides a concentrated signal starting at 100% and extending beyond 130% of threshold intensity. For the AnT, it represents a spectrum of supra-threshold intensities extending from HIIT (110–120%) toward all-out SIT (≥130%). The observed interventions suggest that the adaptive response may further extend into supra-threshold intensities, although the available evidence is insufficient to define an upper boundary or an optimal supra-threshold range. Importantly, implementing these training prescriptions requires identifying the optimal intensity level at which further increases no longer provide additional benefits for each threshold, a topic that warrants further research.

Therefore, although a fundamental three-zone TID model anchored on thresholds offers a useful macro-structure for describing intensity and volume, it may lack sufficient resolution. More granular intensity frameworks (*e.g.*, five-zone models) may therefore better support precise prescription within the adaptive window (Watts et al., 2023; Haugen et al., 2022). Once the athlete has achieved the desired threshold adaptations, training can shift from more granular model to desired fundamental TID-based frameworks for competition or maintenance phases, providing a macro-structure to balance volume, facilitate recovery, and optimize performance while minimizing the risk of non-functional overreaching (Guellich & Seiler, 2010).

While volume-related moderators were not statistically significant, descriptive trends offer hypothesis-generating descriptive observations. AeT adaptations were most pronounced with longer training durations and higher session frequencies (≥52 weeks; ≥5 sessions per week), reflecting the protracted timeline required for structural capillary angiogenesis and other slow structural remodelling processes (Haugen et al., 2022; Brooks, 2018; Muscella et al., 2020). Importantly, total time-in-zone appears to be a more critical factor than total session length for driving these adaptations (Seiler & Kjerland, 2006). In contrast, for AnT, accumulating approximately 30 min per session in the supra-threshold metabolic range appears sufficient to elicit meaningful progression, in line with observations from team sports (Buchheit et al., 2025). AnT improvements were most pronounced in short-term programs (4–8 weeks; ≥3 sessions per week); consistent with the rapid molecular remodelling of lactate shuttling (Brooks, 2018) and a relatively narrow adaptive window. Although these patterns were not statistically significant in our models, likely due to limited power, they closely mirror optimal practices reported in broader literature (Rosenblat, Perrotta & Vicenzino, 2019; Yang, Wang & Guan, 2025). AnT gains declined in longer interventions (+5.8%), reflecting a physiological ceiling likely driven by saturation of lactate transporter expression and intracellular buffering capacity (Svedahl & MacIntosh, 2003). As athletes adapt, supra-threshold stimuli of AnT can lose potency; without upward recalibration, initial supra-threshold workloads effectively revert to ‘at-threshold’ or ‘sub-threshold’ intensity. Given the rapid adaptation observed in short-term interventions (<8 weeks), periodic reassessment using standardized CPET may be warranted for AnT, particularly in non-elite cohorts. For AeT, such adjustments serve more as a milestone check given the slower structural nature of those adaptations. Overall, these findings seem to point out a contrast in adaptive timelines between the AeT and AnT metabolic anchors.

### Limitations and future trials

A key strength of this review is the use of a pre-registered protocol, double-blind screening, and three-level meta-analytic modelling to account for complex data dependencies. Given our sample size constraints, including secondary variables in the formal meta-regression models would have introduced a significant risk of statistical overfitting and generated unstable estimates (Harrell, 2015). Consequently, this focused approach ensures that our conclusions are derived from the most robust evidence within the dataset, identifying intensity as the most consistent moderator of training adaptation.

However, several limitations must be acknowledged. First, the small number of included studies, particularly for the AeT subgroup (*k* = 9), limited statistical power and increased vulnerability to overfitting. Even though additional studies exist, our strict inclusion criteria restricted the final sample size. For the AeT, the ANCOVA model for outcome units revealed perfect confounding and multicollinearity, rendering parameter estimates for speed *versus* power unreliable. Consequently, descriptive trends favouring speed require confirmation in larger future samples. A second fundamental limitation is the lack of standardization in physiological anchors determination methodology. Several criteria exist for metabolic milestones identification, which can result in disparate absolute intensities being labelled as the threshold (Faude, Kindermann & Meyer, 2009; Iannetta et al., 2020). Our ANCOVA models identified a significant bias where LT-based methods yielded larger ESs than VT-based markers (*p* = 0.018). This may reflect greater sensitivity of lactate to training or inherent measurement noise (Faude, Kindermann & Meyer, 2009; Meixner et al., 2025). Nevertheless, the dominance of the intensity stimulus remained robust even after controlling for identification methods, suggesting the metabolic signal of moving ‘above’ an individual anchor outweighs the noise of the criteria used. Thirdly, expressing threshold adaptations primarily in relative terms (%) represents a limitation in interpreting practical significance. Because our primary outcome variable integrated disparate metrics, power, speed, and VO_2max_, the back-transformed percentage changes may mask the varying physiological ‘cost’ and performance impact associated with each unit. Although we utilized a hierarchical rule (power > speed > VO_2max_) to prioritize the most prescriptively actionable outcomes, the lack of standardized absolute reporting across the literature prevents a more detailed assessment of the clinical or performance-related magnitude of these shifts. Furthermore, we did not explicitly categorize studies by work-matching protocols. Because volume-related variables demonstrated weak signal strength in Stage 1 exploratory analysis, they were excluded from formal meta-regression to prioritize model stability. While this suggests that adaptation is more sensitive to intensity than volume, we cannot definitively rule out the residual confounding between intensity and volume. The observed superiority of supra-threshold groups may reflect an integrated response to both higher metabolic signal and a higher cumulative load. Finally, methodological quality was mixed, with 38% of studies demonstrating a high risk of bias and the presence of significant publication bias for the AnT subgroup (*p* = 0.0005) suggesting that the literature itself is a skewed representation of the true effect, likely overestimating the mean improvement due to the non-reporting of low effect and non-significant studies. A further limitation relates to the sensitivity of the search strategy regarding functional anchors and training models. Our search string was designed to identify metabolic transitions (LT and VT) rather than performance-based mathematical models such as ‘Critical Power’ or ‘Critical Speed.’ While these concepts are related, they represent distinct physiological boundaries, and their exclusion was a deliberate choice to maintain the homogeneity of the metabolic outcome variables. Furthermore, while specific terms for training intensity distributions (*e.g.*, ‘polarized training’ or ’threshold training’) were not explicitly included, studies utilizing these models were captured *via* the combination of ’training’ and threshold-specific nomenclature. Nonetheless, we acknowledge that the absence of these terms may have influenced the total number of records retrieved. Finally, the inclusion of both randomised controlled trials and single-group pre-post interventions represents a methodological limitation. While including single-group designs was necessary to maximize the sample size in this specific niche of endurance physiology, it increases the risk of regression to the mean and temporal confounding. Specifically, without a control group, we cannot definitively rule out that a portion of the observed threshold shifts resulted from participant habituation to the testing protocols or seasonal variations.

This review identifies critical research gaps. First, there is a historical asymmetry in which the AnT has been prioritized over the AeT (Binder et al., 2008; Seiler & Tønnessen, 2009). The near-total absence of data on elite athletes and female populations severely limits our understanding of these principles at the highest levels of performance. Future research should prioritize rigorous, work-matched RCTs that utilize standardized identification criteria and non-linear load matching (*e.g.*, TRIMP) to precisely map the dose–response relationship within defined training zones across diverse populations.

## Conclusion

According to this systematic review, the most prominent moderator identified in this synthesis for adaptation seems to be training intensity. Training above, rather than at or below the respective metabolic milestones, was consistently associated with larger threshold adaptations. While participant training status and intervention length were not statistically significant moderators in our formal inferential models, descriptive trends suggest that adaptations are most pronounced when supra-threshold work is applied frequently and over longer durations for the AeT, *versus* concentrated, short-term stimuli for the AnT. However, the evidence is currently insufficient to establish a precise ‘optimal’ supra-threshold intensity or to entirely isolate the independent effects of intensity from total cumulative training load. Accordingly, while the magnitude of the supra-threshold effect is substantial, these results should be interpreted as strong observational trends rather than absolute evidence of causality. Therefore, these findings suggest that individualized supra-threshold training, tailored to the athlete’s current physiological threshold and regularly reassessed over time, may represent a promising approach, although further research is needed to determine the optimal intensity, volume, frequency, and duration according to individual characteristics.

## Supplemental Information

10.7717/peerj.21806/supp-1 Supplemental Information 1 PRISMA checklist

10.7717/peerj.21806/supp-2 Supplemental Information 2 Risk of bias, data exploration, model validation, sensitivity analysis, and supplemental figures

## Additional Information and Declarations

### Data Availability

The following information was supplied regarding data availability: This is a systematic review/meta-analysis. Peric, R. (2025). Systematic review on threshold training raw data [Data set]. Zenodo. https://doi.org/10.5281/zenodo.17523709.
