# Supplementary material for: Moderators of metabolic threshold adaptations: a systematic review and meta-analysis
Source: PeerJ. 2026 Oct 22;14:e21806. doi: 10.7717/peerj.21806 (PMC13695963; doi:10.7717/peerj.21806)
Supplement: Supplementary file 2 [file peerj-14-21806-s002.docx]

**Risk of Bias**

The methodological quality of the 13 included studies showed mixed risk of bias results (Fig. 2). Nearly half of the studies (46.1%) were judged to have a low risk of bias. These studies were characterized by randomized or well-controlled trial designs with appropriate control groups and baseline matching. Five studies (38.5%) were assessed as having a high risk of bias, a decision resulted from their single-group, uncontrolled pre-post design, which cannot distinguish intervention effects from the passage of time or other confounding factors. The remaining two studies (15.4%) presented some concerns, typically due to the absence of a non-exercising control group, which limits certainty about the magnitude of the training effect. A common methodological limitation across nearly all studies was a lack of blinded outcome assessment.

**Data Exploration**

The unweighted descriptive statistics revealed a significantly larger mean % change in the AeT subgroup (+23.1% (14.4%)) compared to AnT subgroup (+9.6% (6.3%)). % change range was 2.1%, +47.9% for AeT and +2.0%, +23.6% for AnT, respectively.

For AeT subgroup, trainings occurring above the AeT (k=3) averaged a +38.7% improvement, which was substantially greater than for those prescribed at the AeT (k=5, +14.7%) or below it (k=1, +18.4%). Untrained participants (k=5) averaged a +26.4% improvement, a much larger gain than that seen in amateur athletes (k=4, +19.0%). Long-term (52 wks, k=2) interventions averaged +32.1% improvement, compared to +17.5% for medium-term (12-18 wks, k=4) and +19.02% for short-term (6-8 wks, k=3) interventions. Cycling-based interventions (k=6) yielded a slightly higher mean effect (+23.7%) compared to running-based interventions (k=3, +22.1%). The single mixed-format study (+40.3%) showed the largest effect, while the eight continuous format studies averaged +21.0%. There were no interval training studies in this subgroup. Average improvement in females (k=6; +26.6%) was higher than improvement reported for both mixed-sex (k=2, +23.2%) and males (k=1, +2.1%). The studies with session durations ≤45min (k=2) showed greatest improvements (+38%), which was considerably higher than those with moderate (>60min) (+22.1%) or long (>120min) (+16.6%) durations. Groups training 5-6 times/week (k=4) averaged +32.6%, compared to +15.6% for those training 3-3.5 times/week (k=5). Studies using LT methods (k=4) reported a mean improvement of +32.5%, which was double the effect reported by studies using VT (k=5, +15.6%). Studies reporting outcomes in speed (k=2, +32.1%) showed larger mean effects than those reporting in power (k=4, +19.0%) or VO_2_ (k=3, +17.5%), suggesting that the choice of outcome unit can significantly influence the observed ES when compared to the exercise modality.

For the AnT subgroup, groups training above the AnT (k=7) averaged a +12.2% improvement, compared to +7.9% for at (k=7) and those at below (k=1) intensities (2.6%). The untrained group improved by +10.3%, amateur group (k=13) by +9.6%, and the elite group by +9.1%. Interval training (k=5) led to a mean improvement of +14.0%, which was more than double that of continuous training (k=8, +7.5%) or mixed (k=3, 7.1%). Cycling interventions (k=10) were most effective, averaging +12.5%, followed by mixed-modality of running and cycling (k=1, +10.3%) and running (k=4, +4.2%). Short-term (4-8 wks, k=9) interventions averaged +10.1%, more effective than medium-term (10-14 wks, k=3, +7.6%) but more than long-term (36-40 wks, k=2, +5.8%) programs. Mixed-sex groups (k=3) showed the largest mean improvement (+16.7%), followed by female-only (k=2, +10.6%) and male-only (k=10, +7.3%) groups. Mean improvements were similar across interval short (+9.4%), medium (<30min) (+8.7%), and long (>60min) (+7.2%) duration categories. Furthermore, groups training up to 3 times/week (k=10) averaged +9%, while those training at a higher frequency (>6 times/week (k=5)) averaged only +6.9%. VT methods (k=7) averaged +12%, better then LT (k=7, +7.2%) and the single both study (k=1, +9.5%). Mean effects were comparable across power (+12.7%) and VO_2max_ (+11.6%), but better than for speed (+4.3%). Across the 24 intervention groups, sub-threshold training ranged from 84% to 90% of the threshold, at-threshold was defined as 100%, and supra-threshold ranged from 110% to 150%. For AnT, supra-threshold intensities (k=7) averaged 124 ± 13% (range: 110–150%), while for AeT (k=3), they averaged 128 ± 19% (range: 110–147%). These ranges informed our decision to model AnT intensity as a continuous variable to explore dose-response trends, whereas the limited number of AeT supra-threshold groups (k=3) precluded reliable continuous modelling.

Based on this systematic exploration, three substantive moderators demonstrated the strongest and most consistent physiological signals across both subgroups, justifying their selection for formal inferential modelling: training intensity, participant status and intervention length (Table 2).

**Model Validation and Sensitivity Analysis**

In our model diagnostics, the Shapiro-Wilk test to assess the normality of residuals was non-significant for both the AeT subgroup (W = 0.971, p = 0.904) and the AnT subgroup (W = 0.932, p = 0.291), indicating normal distribution. For both the AeT and AnT models, all Cook's distances were found to be very small indicating no influential outliers (values < 0.46 for AeT, and < 0.25 for AnT). Both models met assumptions for residual normality and exhibited no undue influence from individual studies or groups.

For the AeT subgroup, the original pooled estimate (in LnRR scale) was 0.191. When removing one study at a time, the pooled estimate remained highly stable, ranging from a low of 0.166 to a high of 0.218. For the AnT subgroup, the original pooled estimate was 0.075. The results were even more robust in this subgroup, with the LOSO estimates varying only within a very tight range from a low of 0.067 to a high of 0.082. The stability of the pooled estimates in both LOSO analyses confirms that the overall conclusions are robust and not driven by any single influential study.

The bootstrap analysis was performed on the intervention length moderator only, due to stability and robustness due to theoretical importance, continuous nature, and consistent data availability.

For the AeT subgroup, the visual inspection of funnel plot displays a reasonably symmetrical distribution of the 9 ESs around the pooled estimate (fig 3). The studies are scattered on both sides of the overall effect line without a clear or systematic pattern of missing studies in any quadrant. This visual impression of symmetry is confirmed by the formal Egger's regression test, which was not statistically significant (p = 0.19). Together, these results provide no evidence of publication bias for the AeT subgroup. In contrast, the visual inspection of funnel plot for the AnT subgroup displays clear visual evidence of asymmetry (fig 3). While the most studies at the top of the plot are clustered relatively symmetrically around the pooled effect, there is a notable absence of studies in the bottom-left portion of the plot. This visual observation is strongly confirmed by the Egger's regression test, which was statistically significant (p = 0.0005).

For the AeT, the bootstrapped 95% CI for the slope of intervention length is [-0.0355, 0.0309] whereas the AnT results reported the bootstrapped 95% CI for the slope of intervention length [-0.0038, 0.0030]. For both subgroups, the 95% CI from both thresholds comfortably include zero. This robust sensitivity analysis confirms that there is no statistically significant relationship between the length of the intervention, as a continuous variable, and the magnitude of the training effect for either AeT or AnT.

**APPENDIX FIGURE 1**

**Figure 1.** Risk of Bias

**APPENDIX FIGURE 2**

**Figure 2**. Funnel plots for risk of bias a) AeT & b) AnT. The symmetrical distribution in plot (a) suggests low risk of publication bias, confirmed by a non-significant Egger's test (p = 0.19). The asymmetry in plot (b), with a lack of studies in the bottom-left quadrant, suggests potential publication bias, confirmed by a significant Egger's test (p = 0.0005).
